# Shades of hope: Marcel’s notion of hope in end-of-life care

**DOI:** 10.1007/s11019-021-10036-1

**Published:** 2021-06-25

**Authors:** Marta Szabat, Jeanette Bresson Ladegaard Knox

**Affiliations:** 1 Department of Philosophy and Bioethics, Faculty of Health Sciences, Medical College, Jagiellonian University, Michalowskiego 12, 31-126 Krakow, Poland; 2 Department of Public Health, University of Copenhagen, Øster Farimagsgade 5, building 10, 1014 Copenhagen, Denmark

**Keywords:** Marcel, Phenomenology of hope, End-of-life-care, Palliative care, Terminally ill

## Abstract

This article examines the compatibility and relevance of Gabriel Marcel’s phenomenology of hope in interdisciplinary research on the role of hope in end-of-life (EOL) care. Our analysis is divided into three thematic topics which examine the various shades of hope observed in Marcel’s phenomenology of hope and in the collection of 20 EOL studies on hope as experienced by adult palliative care (PC) patients, health care professionals (HCP) and parents of terminally ill children. The three topics defining the shades of hope are: the meaning of hope in its dynamic aspects, the dialectics of hope and despair, and the transcendent facets of hope. We analyse how Marcel’s understanding of hope is reflected in EOL studies, and how this perception can enrich the philosophy of PC and significantly deepen and broaden HCPs’ understanding of hope. Our findings prove that despite terminological differences between Marcelian phenomenology and the concepts of hope in the 20 EOL studies, hope emerges as a resourceful movement towards being. Implementing Marcelian hope within communication in EOL care could help in HCPs’ interpersonal approach to patients as his concept harbors a holistic perception of the existential situation of a person. Equally, introducing Marcel’s phenomenology of hope into the clinical encounter could play a beneficial role in improving the ability of patients to adapt to the difficult conditions of their disease and PC treatment.

## Introduction

Life is unpredictable, yet sure to bring challenges that call for an attitude of perseverance, fortitude and humility. Hope becomes significant in healthcare as a care practice and as a moral resource when a deadly disease threathens a life. But what is, essentially, hope and what kind of hope is beneficial in end-of-life (EOL) care? Several studies on patients in the palliative phase of illness confirm the essential possibility of understanding hope as a vital and fundamental act of personal will and as a useful tool in EOL care to cope with overwhelming situations (Bally et al. 2014, Daneault et al. 2016, Granek et al. 2013, Hill et al. 2014, Nierop-van Baalen et al. 2016). The notion of hope in EOL care can be described in many different ways: hope in its semiotic aspect, hope as a phenomenon that emerges from a certain cultural discourse (Daneault et al. 2016) and hope as a variable that can be measured using several scales and according to psychological methods (Feudtner et al. 2010; Kim et al. 2014, Rumpold et al. 2017). Additionally, hope can be studied as a feeling, attitude or even as a decision to maintain positive expectations in relation to one’s circumstances (Bally et al. 2014; Granek et al. 2013; Hill et al. 2014; Misco et al. 2015; Olsman et al. 2014). Finally, hope can be viewed as positive psychological capital and serve as an emotional coping strategy for reducing stress (Folkman 2010).

Parting from these more or less quantifiable perspectives, we observe that Gabriel Marcel (1889–1973) positions hope as a concrete approach to the mystery of “being”. Hope is both concrete, or embodied, and metaphysical to Marcel. Without its metaphysical content, it can be mistaken for the optimism of ‘everything will be all right’ or the desire for something specific. Marcel pays special attention to the phenomenology of hope in his book *Homo Viator. Introduction to a Metaphysics of Hope* (1951) in which he portrays man as a Homo Viator, a wanderer in search of a higher purpose and addresses hope as an ontological phenomenon that has a significant bearing on the formative life journey of an individual in their relationship with others.

At the very outset, it is critical that we underline the differences between three central categories in Marcel’s philosophy, i.e. the levels of “Being”, “being”, and “having”. “Being” with a capital ‘B’ touches a transcendent dimension of human existence that defies logical and intellectualistic comprehension. Being cannot be approached by purely conceptual means as it is a mystery that one can participate in. In our being, according to Marcel, there is an ontological exigence toward something greater than ourselves, our individual being. This Being is not necessily to be understood in a religious way (Knox 2011). Rather, it signifies a depth in our being that surpasses our control and points to the unverifiable realm of Being. However, Being is not something aloof. My personal being participates in Being by way of a concrete approach such as hope through which I am offered existential fulfillment. A concrete approach such as hope uncovers an interpersonal and intersubjective world of “I-Thou” where two or more individuals relate to each other in a non-objectifying manner. The “I-Thou” relationship consists of people being fully present with one another in a living, genuine dialogue, forming a communion. According to Marcel, “horizontal transcendence” keeps a human being at the level of “being” while “vertical transcendence” indicates a connection to “Being” (Marcel 1960a, p. 49; Randall 1992, pp. 285–286). The category of “having” intersects an element of possession and objectification into human relations that can create detachment and alienation from Being and being.

The main thesis of this article is that Marcel’s phenomenology of hope can significantly deepen and broaden health care professionals (HCPs’) understanding of hope. Hope cannot be treated in a narrow sense, i.e. only as a part of a therapeutical strategy or as a useful tool in EOL care, but rather needs to be comprehended in the wider context of a patient’s individual realities in health care, not only holistically but also existentially. Holistic care goes beyond the medical model of treatment to encompass the whole life story of patients and their multifaceted needs. Marcel’s perspective of “Being” and “being” adds an irreductible condition of being human as such, which becomes an essential feature of EOL care, especially at the decision-making level when maintaining hope in patients remains a crucial aspect of such care.

This article analyses Marcel’s phenomenology of hope in the context of 20 studies on the experience of hope among adult palliative care (PC) patients and HCPs as well as parental hope for terminally ill children. It examines the compatibility and relevance of Marcel’s philosophical perspective in interdisciplinary research on hope in EOL care. We discuss how Marcel’s understanding of hope is reflected in EOL studies as well as how his concept of hope can enrich the philosophy of PC, which should be approached holistically in the sense of supporting the physical, psychological, social and spiritual needs of patients as well as their families and friends, not only during treatment but also during the bereavement stage (Mok et al. 2010; Saunders et al. 1995; Saunders 2006; Brown et al. 2016; Friedrichsdorf and Bruera 2018). Through Marcel’s philosophical perspective on hope in the context of EOL care, we look at the important role it plays in interpersonal communication and in forming basic attitudes in relationships between HCPs, patients and their loved ones.

### Methodology

In the article, we reflect on Marcel’s distinction between “Being”, “being”, and “having” from the perspective of hope in EOL care. As a consequence, we follow Marcel’s methodology based on, in Marcel’s own words, “primary” and “secondary reflection (Marcel 1963, p. 75). “Primary reflection” considers any form of existence by objectivizing it in the sense that it reduces existential phenomena to the data on which, for example, scientific knowledge is based. This kind of reflection is adopted in relation both to problems that need to be solved and to definitions designed to calculate the specificity of an issue. At this level of reflection, instead of experiencing a mystery, we measure, calculate, verify, and solve problems. In contrast to “primary reflection”, the second type introduces the existential perspective of “being” and “Being” where it is impossible to reduce human existence to problems or mondane obstacles like not being able to find one’s keys. “Secondary reflection” unifies all forms of knowledge while at the same time giving them insight into human experience in its real and unpredictable form.

The analytical strategy adopted in this article consists of three thematic topics which examine the various shades of hope observed in Marcel’s phenomenology of hope and in the collection of EOL studies. The three topics focus on defining the shades of hope as the meaning of hope in its dynamic aspects, the dialectics of hope and despair, and the transcendent facets of hope. Marcel’s approach to hope functions as theoretical scaffolding for the analysis of the EOL studies revealing the basic components encompassed by his phenomenology of hope. In our study, Marcel’s theoretical framework constitutes the theoretical foundation for the “secondary reflection” while the findings of the EOL studies are analysed on the level of Marcel’s “primary reflection”.

These three fundamental shades of hope were identified after a careful reading of Marcel’s central treatise on hope entitled “A Sketch of a Phenomenology and a Metaphysic of Hope” contained in “Homo Viator. Introduction to the Metaphysic of Hope” (Marcel 2010, pp. 23–61) from the perspective of his other works. Marcel’s conceptualization of hope is discussed in relation to 20 empirical studies on hope in EOL care to explore commonalities and the potentiality of Marcelian hope in EOL care. The research was based on a search for the keywords ‘hope* palliative care’ in the Web of Science Core Collection database conducted on September 13th 2020. The data was then exported to the Excel files. A total of 755 records were collected. Firstly, an automated search procedure was applied for duplicates. Because the automated function did not manage to detect all the duplicates, the first author (FA) conducted a manual searched for duplicates. Secondly, the selection process was based on four criteria: (1) publication type, such as qualitative research, interviews and systematic reviews conducted with the aim of extracting personal experience, which is an important feature of Marcel’s phenomenology of hope; (2) research topic, such as hope in PC; (3) Marcel’s conceptualization of hope-related content, and (4) all the studies included in the research were in English. Doctoral and master’s theses or dissertations were not considered because we expected that the most rigorous of them would have already been published as articles. Books and book chapters were also excluded due to the size of the article and the goal of extracting the existential dimension of hope in PC. Finally, commentaries and editorials were eliminated because most of them were not based on empirical foundations. In the case of the second selection criterion, only articles concerned with the study research topic were taken into account. The articles were summarized and, subsequently, elements of three thematic topics in Marcel’s phenomenology of hope were extracted. In the preliminary phase, the FA first screened the records in accordance with the eligibility criteria (EC) and 22 studies were selected for the further analysis. Then the second author removed two articles that did not meet the EC. Finally, 20 studies were selected for the analysis (see: The table of the studies on hope in end-of-life care examined in the paper). All the articles included were considered to be of sufficient quality, based on the peer review process and on the academic reputation of the journals.

**Table:** The table of the studies on hope in end-of-life care examined in the paper

Authors	Methodology	Findings
Bally et al. (2014) (Canada)	Charmaz’s constructivist grounded theory (ChCGT)	Parental hope (PH) is described as an essential, powerful, deliberate, life-sustaining, dynamic, cyclical process rooted in time through the actions of accepting reality, establishing control, restructuring hope, and purposive positive thinking. The research reveals several aspects of PH such as the calming and strengthening function of hope, facing new challenges, avoiding the loss of hope, facing fear and despair
Bally et al. (2018) (Canada)	Review qualitative studies	Parents experience was described as a dual reality in which fighting for survival and recognizing the threat of their child's death were daily challenges. 3 key processes emerged: the devastation of living with uncertainty, the emergence of hope, and moving forward
Clayton et al. (2008) (Australia)	A systematic review	Findings suggest that balancing hope with honesty is an important skill for health professionals (HPs). Many patients seem to be able to maintain a sense of hope despite acknowledging the terminal nature of their illness. Patients and caregivers mostly preferred honest and accurate information, provided with empathy and understanding. HPs need to recognize this spectrum of hope and appreciate that patients may simultaneously hope for ‘cure’ while acknowledging the terminal nature of their illness. HPs may help patients to cope with their terminal prognosis by exploring and fostering realistic forms of hope that are meaningful for the particular patient and their family
Daneault et al. (2016) (Canada)	ChCGT	The coding procedure revealed 7 categories of hope: (1) an irrational; (2) initial hope for miraculous healing; (3) as a phenomenon that changes over time; (4) hope for prolonged life when there is no further hope for cure; (5) hope for a good quality of life; (6) a lack of hope for some when treatments are no longer effective in curbing illness progression; (7) hope as enjoying the present moment and preparing for the end of life
Eliott and Olver (2009) (Australia)	Interviews according to the interview protocol, discursively analyzed responses	3 type pf hope were identified: (1) essential to, and for, life; (2) hope, life, death, and others; (3) hopes changing during (or in) life
Granek et al. (2013) (Israel, Canada)	ChCGT	2 categories of hope emerged: (1) future-oriented hope and (2) present-oriented hope. Additional subcategories were identified under each main categorysuch as: (1) hope for a cure and treatment success, hope for the child’s future, hope for a miracle, hope for more quality time with child. (2) Hope for day-to-day/moment-to-moment, hope for no pain and suffering, hope for no complications
Lou et al. (2015) (Republic of China)	Colaizzi’s descriptive phenomenology (CoDPh) and Husserl’s phenomenological approach in data analysis	5 main topics were established: (1) losing hope for a cure; (2) encountering death; (3) establishing a protective role toward the child; (4) the intertwining chaos; (5) strengths of family life, contending with death. Within the issue (1) the authors indicated 3 subcategories: (a) confirmation of fear and desperation concerning recurrence of the illness; (b) the hope for an extended life; (c) the adjustment of expectations toward comfort care
Mok et al. (2010) (Hong Kong)	A qualitative study, the methodology was based both on Husserl’s and Heidegger’s approaches	4 meanings of hope: (1) expected hopelessness/HCPs expected hopelessness or despair and were surprised if patients did not express such feelings/; (2) a dynamic process of hope; (3) hope-fostering strategies/the patients’ adaptation to EOL issues/; (4) peace as the ultimate hope
Mrig and Spencer (2018) (USA)	An anthropological investigation into the attitudes of oncologists and primary care doctors toward the decision to stop active treatment and begin palliative treatment, a qualitative research was conducted using data collected over interviews with doctors, which was then transcribed and reviewed by each author of the paper	The authors argue that hospice care is underutilized due to the ‘fighting or giving up’ attitude shown by doctors themselves and that the culturally conditioned metaphor of ‘war’ can be found to underly this approach. Cancer treatment is understood within this metaphor according to which the patient is supposed to play the role of soldier. The oncologist’s role is that of commander, planning how to win the battle. The doctors’ statements reveal the strong link between their hope and optimism in the sense of fighting, regardless of the price patients and their close ones might pay—not only in the case of failure, but also during aggressive treatment
Nafratilova et al. (2018) (Indonesia)	CoDPh	The parents’ religious attitudes, including their hope for a miraculous cure for their children and their need to surrender to God
Nierop-van Baalen et al. (2016) (Belgium, Ghent)	The study contains a secondary analysis of data obtained in a series of small studies on living with cancer with a short life expectancy (between 3 and 12 months)	The meaning of hope is related to the importance of the object it is attached to, rather than to a real chance of achieving this object. A dual function of hope: patients hope because they cannot forsake it and because they benefit so much from it. Patients use different strategies to increase their hope, described as the ‘the work of hope’. A better understanding of the work of hope can lead to better psychosocial support by HPs
Olsman et al. (2014) (The Netherlands)	The interpretative synthesis consisted of a quality assessment and thematic analysis of included articles	The majority of these 31 articles described perspectives of nurses or physicians. 3 perspectives on hope of palliative care patients were found: (1) realistic—hope as an expectation should be truthful, and healthcare professionals focused on adjusting hope to truth; (2) functional—hope as coping mechanism should help patients, and professionals focused on fostering hope, and (3) narrative—hope as meaning should be valuable for patients, and healthcare professionals focused on interpreting it
Rallison and Raffin-Bouchal (2013) (Canada)	The methodology of hermeneutic phenomenology	The main outcome of the study revealed 3 themes: (1) the parents’ experience of predictability and unpredictability; (2) the complex and continuous care provided in their homes; (3) the spiritual journey of the families
Reinke et al. (2010) (USA)	The classic version of grounded theory, audiotaping and transcribing semi-structured interviews with nurses caring for patients with advanced chronic obstructive pulmonary disease or cancer	3 themes: (1) maintaining patient hope; (2) providing prognostic information; (3) coordinating the provision of prognostic information with physicians
Robinson (2012) (Canada)	Audio-recorded interviews, thematic analysis conducted using constant comparison	Hope is a significant theme in the advanced care planning (ACP). 3 themes were identified: hope is multifaceted, hope for a cure is well considered, and hope is resilient and persistent. The seeming paradox of hoping for a cure of an incurable cancer did not interfere with the process of ACP
Rempel et al. (2012) (Canada)	Glaser’s grounded theory and ChCGT	‘Parenting under Pressure’ process consists of 4 phases: (1) realizing and adjusting to the inconceivable; (2) growing increasingly attached; (3) watching for and accommodating the unexpected; (4) encountering new challenges
Roscigno et al. (2012) (USA)	Semi-structured interviews, data came from a longitudinal multiple case study investigation	Parents relied on hope as an emotional motivator, whereas most HPs described parents’ notions of hope as out of touch with reality. Parents perceived that such divergent beliefs about the role of hope negatively shaped communicative interactions and reduced trust with some of their providers
Szabat (2020) (Poland)	Initial and focused coding using ChCGT and elements of CoDPh	The experience of PH consists 3 aspects: hope for the longest possible presence of a child with a family, hope for control over situations, pain, and symptoms, and existential facets of hope
Timmermann et al. (2015) (Denmark)	A phenomenological-hermeneutic study based on Paul Ricoeur’s hermeneutic	The findings reveal an influence of nature/weather on patients’ behavior. The analysis revealed 3 themes: (1) the experience of inner peace and an escape from negative thoughts; (2) the experience of a positive mood; (3) hope and the experience of good memories
Vachon et al. (2009) (Canada)	A conceptual analysis	11 dimensions for the concept of EOL spirituality: (1) meaning and purpose in life; (2) self-transcendence; (3) transcendence with a higher being; (4) feelings of communion and mutuality; (5) beliefs and faith; (6) hope; (7) attitude toward death; (8) appreciation of life; (9) reflection upon fundamental values; (10) the developmental nature of spirituality; (11) its conscious aspect

Our study has a number of limitations since only documents published in English were considered eligible. This limitation may narrow down the results, since cultural, psychological and ethical issues associated with hope appear to be clinically sensitive, and it is possible that not all forms of hope have been discussed in English. However, the purpose of our study is to show the existential aspects of hope in EOL care by using Marcel’s methodology and 20 selected studies were deemed a sufficient number for this purpose.

It is important to stress here that the aim of this article is not to present as many studies on EOL care as possible but rather to identify the fundamental connections between Marcel’s concept of hope and the findings in these 20 empirical studies. In addition, for the purposes of the present article, we decided to select studies that provide the most exhaustive accounts of the specific commonalities important for hope in EOL care, taking into consideration the experiences of patients and HCPs. According to Marcel, a philosophical approach to hope is defined by a personal, embodied experience, which is the reason for selecting qualitative studies in which personal statements are treated as the starting points for coding, analysis and theory building. Following Marcel’s method, in which he grounded his reflections on real-life examples, we focused our search on personal experiences on hope. Furthermore, similarly to Marcel, who posited the idea of refreshing philosophical language with ordinary vocabulary (Marcel 1965b, p. 158), many contemporary studies on hope adopt qualitative methodologies with a view to preserving the unique, irreplaceable experiences of a human being.

## The dynamic aspect of hope in Marcel’s phenomenology and end-of-life studies

At the beginning of his essay “Sketch of a Phenomenology and a Metaphysic of Hope”, Marcel recognizes the experience of hope as the point of departure for further analysis of hope, claiming that the personal aspect of hope finds expression through the utterance “I hope…” in the first-person singular (Marcel 2010, p. 23). However, for Marcel, hope stems from the mystery of Being within which there exists a bipolarity between the individual and transcendence. In the act of transcendence, hope connects personal experience with “being”. In this sense, hope springs from a reality well beyond human control, yet it is concrete and accessible. A mystery is unexplainable and inexhaustible and not of human making. Through hope, in Marcel’s metaphysical understanding, a deeper experience of human life is made possible. In fact, hope is the gateway to a fulfilled human life. In order to fully grasp how this is possible, we need to better understand the disposition of Marcelian hope.

Marcel differentiates between “I hope…” and “I hope that”. The term “I hope…” indicates a general, undefinable disposition with no determinate objects of wants. The sense coveyed by the utterance “I hope that” concerns the expectations or wishes of a specific person. Marcel’s differentiation between “I hope…” and “I hope that” points to the origins of hope—a modality of “Being” and “being”. In Marcel’s phenomenology of hope, we observe energetic facets of hope which are modified by such an experience. The first-person singular “I hope…” and “I hope that” indicate a highly private state of individual existence. Similarly, the expression “hope in” reveals in its interpersonal background the dynamism of hope (Marcel 2010, p. 49).

In French (the language in which Marcel wrote) the term hope takes two forms: a noun and a verb. The noun “l’espoir” is related to the prepositions “de” (l’espoir de quelque chose or l’espoir de faire quelque chose.), “dans” and “avec” (dans or avec l’espoir de quelque chose). The verb “espérer que” means “to hope that”, “espérer avoir quelque chose” or ‟espérer avoir de quelque choseˮ indicates “to hope for something” (Collins Dictionary 2021; Cambridge Dictionary 2021). Both forms are connected with the central categories of Marcel’s philosophy, i.e. “being” (e.g. c’est mon espoir) and “having” (e.g. avoir de l’espoir). In his work entitled “The Existential Background of Human Dignity” Marcel underlines the fact that “the Being which is meant in such expressions as ontological weight or stake must be understood as a verb and not as a noun” (Marcel 1963, p. 77). Based on this statement, it is clear that hope in Marcel’s thought should be discussed with an emphasis on our being as an active means of relating to the self and the world.

Moreover, Marcel attaches a certain value to the expression “hope in” vis-à-vis human relationships (Marcel 2010, p. 49). “Hope in” somebody or something means trusting that one’s attitudes or actions will be compatible with our predictions, and also that something we “hope in” will accord with our expectations. Hope understood in this way relies on people’s altruistic attitudes towards one another in the sense of the mutual interactions between them that sustain the very existence of hope. This context of interpersonal meaning warns us of the danger of human relationships slipping into some kind of business transaction model deprived of deeper connections between people. Hope is described by Marcel as a creative process that paves the way for acceptance of difficulties and helps turn them to our benefit. Marcel refers to this as the “*domesticating* of circumstances” (Marcel 2010, p. 34). Hope as an exchange based on altruistic motivations between the one who gives and the one who receives support from various sources, especially spiritual ones, both strengthens interpersonal relations and enriches the spiritual experience of hope (Marcel 2010, p. 44). This sort of hope can help us reject feelings of internal determinism caused by difficult circumstances, and in turn overcome our despair and restore our sense of security (Marcel 2010, pp. 33–35).

The significance of preventing human interaction from being reduced to a form of economic exchange, and a human from being becoming a mere object of economic activity suggests a mysterious connection between the psychical and psychological sides of hope (Marcel 2010, pp. 49–50). Analogous to the notion of light as a source of illumination without becoming an object of light, hope produces hopeful and personal layers of hope but its nature remains mysterious without being objectified. Hope shows the way in the same manner as the soul directs the body. The idea of being guided by the light of hope is expressed in the following words: “By a paradox which need surprise only the very superficial thinker, *the less life is experienced as captivity, the less the soul will be able to see the shining of that veiled, mysterious light*”. (Marcel 2010, p. 26). Prior to this passage, the philosopher referred to Plato’s path, according to which light is more visible in the darkness. Focused around the idea of the dialectic of light and darkness, Marcel recognizes the existential facet of human existence as being “subject to hope” (Marcel 2010, p. 26). Alluding to Plato’s allegory of the cave and the poor condition of a human being living among the shadows, Marcel talks of a human’s inability to know things in a way that can be fully expressed.

Eliott and Olver (2009) observed in their study that when hope was used as a noun, it referred to “an objectively existing entity, centred on the hope of a cure, and associated with a negative future that was beyond the patient’s control. Hope as a verb was typically seen subjectively as determined by the patient, referencing what was personally meaningful, and associated with the depiction of a future with positive aspects”(612). Moving to another quality of hope, Eliott and Olver postulate that “a possession of the patient is its constitution as a thing” and “persons specifically referred to hope as quantifiable, as something that could be there (in varying amounts) or not, or as something that people might have (to varying degrees) or not. Expressed in various ways (e.g., “some hope,” “a lot of hope”), this quantified hope was frequently associated with the possibility of a cure, of getting better, and was thus deemed of vital personal import to patient welfare” (Eliott and Olver 2009, pp. 617–618). In the same study, the authors also analysed the phrase “hoping to live (sometimes in the context of a cure, sometimes not), often using terms such *as longer*, *as long as possible*, or *as long as I can*” (Eliott and Olver 2009, p. 621). In another study, Nierop-van Baalen et al. (2016) report that in uncertain situations patients “use the word ‘hope’ as a noun only to indicate a static situation as in ‘I have lost all hope’” (p. 573). Nierop-van Baalen et al. observe that the category of “my hope” they introduced in this study, “is most discordant with the medical facts” (Nierop-van Baalen et al. 2016, p. 574). This means that a repudiation of the objective fact of death can also be an expression of the act of being “against” reality, which is too overwhelming to deal with at a particular moment.

Rallison and Raffin-Bouchal (2013) revealed how parents described the experience of taking care of their seriously ill children. In their study, we encounter the metaphor of “living in a fog” (p. 197) as a visual depiction of their difficulties in exerting control over their circumstances. Another metaphorical description attempting to grasp the fluctuation of the care process is the notion of living “filled with ups and downs” (Rallison and Raffin-Bouchal 2013, p. 197). This latter metaphor and the similarly expressed “roller coaster ride” appear quite often in studies on parental hope and their experience of caring for their seriously ill children (Bally et al. 2018, p. 92, Szabat 2020, p. 5). Moreover, near-death and grieving experiences are described as “being in the in-between” (Rallison and Raffin-Bouchal 2013, p. 197), signalling the uncertainty of daily life as well as the permanent duality of being near death, which families must cope with every day. This research also discloses the dialectic of the gift in the spiritual aspect of parental hope, the duality of joy and sorrow and a deeper sense of the care process (Rallison and Raffin-Bouchal 2013, pp. 201–202).

Mrig and Spencer (2018) intended their study as an anthropological investigation into the attitudes of oncologists and primary care doctors towards stopping active treatment and beginning PC. The culturally conditioned metaphor of “war” can be observed underlying this approach. Cancer treatment is understood in the context of this metaphor, according to which the patient is supposed to play the role of a soldier. The oncologist’s role is that of a commander responsible for planning how to win the battle. The doctors’ statements reveal a strong link between their hope and optimism in the sense of conitnuing the fight regardless of the price patients and their loved ones might have to pay—not only in the case of failure but also during the course of aggressive treatment (pp. 110–112). Throughout the study, one important remark can be noted in the doctors’ approach itself—using the metaphor of “war” has several consequences, including that of subordinating the interests of patients to a certain “political economy of hope” (p. 107) which is a highly sensitive issue in medical ethics.

If we read the cited studies through the lens of Marcel’s notion of hope we see that the dynamic aspect of hope is prevalent. The form of hope as a noun as employed by Eliott and Olver (2009) and others shows the possibility of hope’s objectivization, which can be overwhelming for patients. Marcel’s concept of “having” seems to explain why this process of reduction may be so terrifying in the context of personal experience. “An objectively existing entity” such as that described by Eliott and Olver (2009), becomes hope beyond human existence but not in the sense of “being”. Conversely, hope as a thing does not offer any possibility of dynamic change. This could be interpreted as the attempt to “domesticate” (Marcel 2010, p. 34) their hopes and try to control them even when they remain illusory and seem unreal. The act of hope makes it possible to overcome reality and pave the way for certain possibilities that elude human cognition. In Marcel’s thought, the level of “Being” justifies the ontological position of hope which constitute the basis of a human being’s existential experience. Without this perspective, personal existence would be reduced to the level of “having”.

## Marcelian dialectic of hope and despair in end-of-life studies

Adversity is built into the fabric of human life, opening the individual to the risk of despair. A person is posited “within the framework of the trial” (Marcel 2010, p. 24) and hope is the response to this trial that life throws at us. The nature of the trial is highly personal and may have both a positive and negative impact on one’s life. Dialectically, hope seems to be related so deeply to despair that the other side of hope—dejection—can initiate the destructive processes of despair. Within this process, it is possible to observe that “hope likewise is liable to degradations” (Marcel 2010, p. 34). Marcel also postulates the importance of taking a risk in hope (Marcel 2010, pp. 48–49). The necessity of taking a risk in hope appears not only to be an indispensable part of the trial but can also be understood as an important aspect of personal experience.

The experience of hardship leaves the individual in a state of mind that may culminate in either hope or despair. As we heard, Marcel’s phenomenology of hope reveals an essential connection between hope and despair according to which hope is not possible without the temptation to feel despair (Marcel 2010, p. 30). Hope presupposes the threat of despair. They are two poles of perpetual oscillation. Where hope signals vitality and openness, despair beckons passitivity and closes the human self on itself. The connection between hope and despair is evident in various EOL studies with their emphasis on the positive function of hope in relation to personal integrity. According to research conducted by Nierop-van Baalen et al. (2016) “People hope because they have no other choice. Without hope life would have no quality or would be unbearable” (p. 573). This statement is helpful for understanding why hope appears so indispensable to personal experience. In the study, the authors provide examples of hope, which they understand as being attached to certain objects, such as the hope that life can be prolonged, the hope that the patient’s condition will improve, that the patient will have a better quality of life or a peaceful death (Nierop-van Baalen et al. 2016, p. 573). The authors refer to hope as “a verbal phenomenon”, which “exists solely when expressed either to oneself or to others. Hope is subjective: it expresses the stance a person takes towards future events that concern them” (Nierop-van Baalen et al. 2016, p. 576). The researchers point to the need for patients and their carers to work on their hope, to come up with appropriate hope strategies to ameliorate the strength of their hope. They describe hope as such a positive phenomenon that it can be used to deal with illness in PC treatment. The study by Rempel et al. (2012) examines parental pressure when caring for young children with life-threatening congenital heart disease. The authors view the process of providing care under pressure as consisting of four phases: “(1) realizing and adjusting to the inconceivable; (2) growing increasingly attached; (3) watching out for and accommodating the unexpected; and (4) encountering new challenges” (pp. 619, 622–627). In both of these studies, we observe efforts made to describe how hope can be controlled using communication tools to improve patients’ quality of life. The mystery of hope is hidden somewhere at the subjective level when the authors, similarly to Marcel, postulate a special category of “my hope”, which encompasses a person’s “deepest desires”, which can be independent of medical facts—patients hope for a real possibility of recovery. As the researchers state: “It is a very personal hope that can barely be communicated and is cherished in silence”(Nierop-van Baalen et al. 2016, p. 574). Thus, as we can also see in the definition provided by Nierop-van Baalen et al. (2016, p. 573), hope is implicitly present as a deeply personal experience.

Another such example is a study of a family’s experience of caring for a child and/or teenager in PC. The fluctuation between hope and hopelessness is depicted in the study in the following way: “the process comprises four sub-processes that represent the symbolic significance of the experience for the family of the child and/or teenager in palliative care, in a context of loss, grief, uncertainty and search for quality of life” (Misco et al. 2015, p. 563). Each identified sub-process resulted in the following categories: seeing one’s life shattered, managing the new condition, acknowledging palliative care and relearning to live. The other study discloses three themes: maintaining a patient’s hope by understanding their specific situation, concentrating on the patient’s quality of life and building trust in the patient; providing prognostic information (assessing patient knowledge and following a patient’s lead); providing prognostic information in coordination with doctors (Reinke et al. 2010, pp. 985–988). Another example can be found in the hermeneutical and phenomenological approach which reveals four meanings of hope: expected hopelessness, a dynamic process of hope, hope-fostering strategies and peace as the ultimate hope (Mok et al. 2010, p. 877). The first meaning of hope concerns the patients’ response to palliative care. While HCPs expected to see an absence of hope, they were surprised if patients did not express hopelessness.

When it comes to the dialectic of hope and despair, a loss of hope is highly delicate issue. The temptation to feel despair based on personal experience is situated, as Marcel postulates, “within the framework of the trial” (Marcel 2010, p. 24). Hope is our stake in maintaining a personal openness to experiences where the temptation to despair would seem to be an easier option than keeping hope alive. However, Marcel’s differentiation between ‘I hope’ and “I hope that” indicates dynamic aspects of hope in that the loss of one kind of hope does not exclude others that are still possible in one’s existence (Marcel 2010, p. 26). The most important lesson to be drawn from Marcel’s reflections is that we should take a risk in hope whenever it is possible.

One example of “losing hope in a cure” can be found in a study by Lou et al. (2015), in which the authors identified three subcategories: confirmation of fear and desperation in the recurrence of an illness, the hope for an extended life, and an adjustment of expectations towards palliative care. This theme is described through a mother’s concerns for her child’s well-being duringend-of-life care. The feelings accompanying palliative treatment include the fear and uncertainty experienced by mothers (Lou et al. 2015, p. 300). One researcher confirms that the uncertainty surrounding hope is rooted in the existence of multifaceted hope (Robinson 2012, p. 75). Moreover, as we showed in the examples above, some forms of hope can be shared by the same group of patients and their loved ones or HCPs, but there are exceptions, such as in Robinson’s study, in which patients diagnosed with advanced lung cancer and their significant others or loved ones (nine family dyads) were interviewed regarding their experiences of hope in advance care planning (ACP)—all of the dyads hoped for as much time together as possible and only one of them hoped for the development of experimental treatment (Robinson 2012, p. 78). The above-mentioned examples and forms of hope have one thing in common, namely that taking a risk in hoping is necessary for highly personal reasons in order to overcome uncertainty, fear and other difficulties.

At the same time, the loss of hope is associated with personal hesitation between hope and its rejection in the face of all the information we have regarding our present and future situations. One strategy for maintaining hope as a possibility seems to be to avoid problematic or detailed information. According to a systematic review of the theme of communicating with terminally ill patients and their families, the majority of patients and caregivers “mostly preferred honest and accurate information, provided with empathy and understanding” while “a minority of patients and caregivers avoid detailed information to preserve hope”. Accordingly, a minority of HCPs “avoid giving information to promote hope” and some patients “deliberately avoided inquiring about progress or symptoms in order to maintain hope for the future, even when they suspected that this was false hope” (Clayton et al. 2008, pp. 641, 643). Human fragility in this case appears to be strongly related to personal resilience and an ability to cope with overwhelming situations. What is more, according to Marcel's categories, the choice between losing and maintaining hope is made at the level of “being”, which implies the impossibility of treating hope only as a useful tool for controlling stress or maintaining it as a safeguard.

As we approach the end of this subchapter and consider the issue of providing information on vulnerable EOL care issues, Marcel’s phenomenology of hope can inspire us in two ways. First, his distinction between optimism and hope (Marcel 2010, pp. 28–29) indicates how the truth should be weighed in the communication process, bearing in mind that false optimism may deprive patients and their families of the opportunity to make their own choices and implement their own plans in EOL care. Second, Marcel’s contrast between “having” (connected to the problematic sphere of existence) and “being” (connected to the mysterious level of being) highlights the fact that personal decisions need to be understood from the perspective of “being” and not simply in terms of a problem to be solved. In EOL care, transmitting and receiving unfavourable information poses a considerable challenge to both HCPs and patients together with their close ones. The authors of the systematic review declare that the studies they included in their search “identified hope as an integral component of the discussion regarding both prognosis and EOL issues […], and suggested that the way this information is delivered is as critical as the content” (Clayton et al. 2008, p. 655). Additionally, they developed strategies that can be helpful for both patients and HCPs so as to ensure proper communication on EOL issues. The authors’ recommendations include tailoring information to individual preferences, including with regard to ACP and other possible treatments, an honest and open discussion between patients and HCPs as well as reassuring patients and their loved ones that effective support and care will be provided throughout the entire treatment process (Clayton et al. 2008, p. 657). What is more, another study on hope among PC patients from an HCP perspective indicates three possible approaches to underlying communication strategies: realistic, functional, and narrative (Olsman et al. 2014, p. 61). The findings confirm that sensitive issues not only require ensuring the right atmosphere and attitudes in communication between HCPs and patients, but also an appropriate vocabulary, including names, epithets, metaphors, phrasal verbs, etc. From a functional perspective, HCPs underline the positive impact that hope has on the way patients deal with diagnosis, treatment and care. The narrative perspective can be helpful in fostering values, beliefs or personal goals important to patients and their close ones (Olsman et al. 2014, pp. 61, 66). All three perspectives of hope can be also used to strengthen any hope strategies.

These three helpful strategies could also find inspiration in Marcel’s objective kinds of judgment. The first kind of judgment addresses objective facts that can be verified. Good examples might be a personal illness or somebody’s death—they are impossible to deny because of actual symptoms or due to the death being confirmed by doctors. The second kind of judgment comes closer to “the root” of personal “objective judgment” and it is discussed using the example of a mother who has lost her son and yet who still believes that he will come back to her and refuses to accept the objective fact of his death confirmed by witnesses. In such a scenario the philosopher poses the following question: “a mother who persists in hoping that she will see her son again although his death has been certified in the most definite manner by witnesses who found his body, buried it, etc. Is not the observer justified in saying that there are no reasons for hoping that this son is still alive?” (Marcel 2010, p. 59). This root of personal objective judgment is captured by Marcel in the phrase “to say to love, against all hope” (Marcel 2010, p. 56). Thus, hope can be related not only to despair but also to love which is as difficult as hope to define. Both the functional and narrative strategies should take into account highly personalazed situations and the vulnerability of the person to whom the information is addressed—to use Marcel’s terms: the mystery of the human being deserves to be taken into account as an important aspect in the communication process.

## Transcendent aspects of hope in end-of-life studies

Marcel employs poetic phrases such as “the availability of a soul” to describe the intimate and, at the same time, spiritual aspects of hope. This phrase seems to be compatible with the metaphor of hope as a light in the darkness. In the essay mentioned above Marcel asks: “But with what kind of hope are we really concerned? What exactly is its object?” (Marcel 2010, p. 26). The answer guides us towards the transcendent aspect of hope which appears as both discursive and non-discursive practices that overcome the idea of interpersonal communication and ascend to a higher level of experience, for example in the form of a communion. This transcendent aspect of hope most likely explains Marcel’s scepticism regarding both physical and psychological theories that seek to explain the phenomenon of hope. He claims that hope is associated with communion in the sense that feelings of personal despair and solitude are enlightened by transcendent hope (Marcel 2010, p. 52).

From Marcel’s point of view, it is possible to add that retaining a hopeful state of mind requires the ontological possibility of believing that hope strengthens personal integrity and allows one to overcome negative emotions, perceptions, and attitudes. The ontological aspect of hope can reveal itself in its absolute dimension, “transcending all laying down of conditions” (Marcel 2010, p. 41). In this meaning, hope may be characterized as a memory of the future in the sense of a reunion, a recollection, a reconciliation with the past, the present and the days to come (Marcel 2010, p. 47).

Marcelian hope focuses on what is beyond my control, connecting with a transcendent dimension that links directly to the realm of the mystery of “Being” and “being”. Transcendence does not mean to transcend experience, but rather refers to unverifiable, yet meaning-making aspects of existence as well as to participation in the question of being. This participation cannot be separated from engaging in intersubjectivity. The transcendent dimension of hope has a vertical and horizontal feature that is analogous to the kinds of hope found in EOL studies. Marcel gives us examples of both forms of transcendence and relates these forms to faith, hope, love, prayer, an absolute Thou or artistic creation—these dimensions reveal a “vertical transcendence” (Marcel 1967, pp. 22–23). “Horizontal transcendence” is grounded in “vertical transcendence” (Marcel 1967, p. 257). “Vertical transcendence” allows human beings’ to shift attention towards the possibility of transcendence in the sense of existing within the realm of metaphysics or the meta-problematic. “Horizontal transcendence” reflects human beings’ intersubjective relationships, ranging from mere communication to genuine communion, from causal encounters to friendship or love.

Daneault et al. describe hope as a “radar guiding a ship on a stormy sea” (p. 651). Like a light, hope determines the possibility of believing in a miraculous cure, even if the probability of effective and successful treatment might objectively be very low (Daneault et al. 2016, p. 651). In addition, several studies show that some patients in EOL care still hope for a miracle (Eliott and Olver 2009, pp. 624–626; Granek et al. 2013, p. 2440; Nafratilova et al. 2018, pp. 127–128; Nierop-van Baalen et al. 2016, p. 574; Rallison and Raffin-Bouchal 2013; Mrig and Spencer 2018). Researchers admit that unrealistic hope, such as hope for a miracle, hope for God’s intervention or hope for a new miraculous treatment in EOL care hold a unique status for patients—children, adolescents, adults (including the elderly)—who can harbour unrealistic forms of hope.

The concept of a “miraculous cure” seems to manifest something that Marcel calls “the gulf between the visible and the invisible” in the case of personal hope at the vertical level where the idea of miracle belongs to a dimension higher than the mortal (Marcel 1965a, p. 79). It is possible to have faith in miracles but impossible to prove or to know that they could happen. On the one hand, hope is invisible and its source essentially unknown, which underlines its mysterious status. On the other, it is possible to experience its outcomes.

In our selected empirical studies “vertical transcendence,” as understood by Marcel, is evident especially in research on the spiritual aspect of hope in EOL care. In one such research project the authors explain: “Transcendence also emerged as a significant component of spirituality. This theme includes all items that relate to a belief in, faith in, or feeling of communion with a Divine or a higher being. It refers to a dimension that transcends the physical, social and material world.” (Vachon et al. 2009, p. 55). Another important aspect of “vertical transcendence” is described by the researchers as “a feeling of communion”, and they go on to claim: “This feeling of communion can be felt with the self, with nature or the environment, with God or the Universe, within interpersonal relationships and even with things. This feeling of mutuality could be defined as something that fulfils the self and that implies a feeling of not being alone” (Vachon et al. 2009, p. 55). From the perspective of spirituality, hope can also be associated with resurrection, which can play an important role in a family’s beliefs and can be linked to such concepts as a better world and immortality.

Marcel’s understanding of the transcendent act of hope in its horizontal aspect is expressed in Roscigno et al.’s study (2012) with the phrase “holding on to hope”. Via different objects such as “past experience, trusted friends or family, magazines, television shows, the internet, and other parents who had been through similar experiences” (p. 1238) (including a diagnosis and information on further treatment), each individual parent, both parents or the family as a whole must overcome their prejudices to palliative treatment or obstacles as they prevent them from holding on to hope. Based on this study, we can observe in what way horizontal transcendence can enrich the intersubjective experiences of both parents at risk of delivering an extremely premature infant and the HCPs taking care of them. Roscigno et al. state that, on the one hand, hope gave parents “the emotional energy to cope with recommended treatments meant to enhance the outcome of the mother’s pregnancy, to make plans for the birth of their baby, and to cope later in the NICU”^1^ (p. 1238). HCPs signalled that the degree and type of hope experienced depended on the child’s prognosis and how much parents could absorb and understand the information they received regarding medical procedures (Roscigno et al. 2012, p. 1240). Another study of Bally et al. (2014) confirm that to facilitate parental hope, which is indispensable to ensure high quality care for their children, parents must be adequately informed of their child’s health, be provided with an opportunity to acquire knowledge, be in contact with others and be ‘in the loop’.

The horizontal form of transcendence can be experienced not only in interpersonal communication but also in contact with nature. In a phenomenological-hermeneutic study of twelve patients carried out by Timmermann et al. (2015), the authors demonstrated how nature impacts on a patient’s well-being. In the first phase of the study, the findings highlighted the influence of nature/weather on patient behaviour, e.g. patients valued sunny weather or the opportunity to see nature, while on cloudy days they remained in their rooms, in bed, etc. (p. 429). The second phase of the analysis revealed three themes: the experience of inner peace and an escape from negative thoughts, the experience of positive moods and hope, and the experience of pleasant memories (pp. 430–431). Positive thoughts and pleasant memories were connected with different forms of nature and exposure to sunlight. The opportunity to be close to nature helped patients avoid worrying about their illnesses and provided distractions from negative thoughts and feelings. Additionally, nature turned out to be a source of joy, hope and pleasure as well as a calming force (p. 430). The final phase of this comprehensive understanding claims that “sunlight or daylight streaming through the windows could bring a sense of hope in the middle of despair” (p. 431). The study teaches us that interpersonal support from HCPs (knowledge, proper communication, experience, appropriate treatment), from family and loved ones should be combined with the need to incorporate a patient into their environment to strengthen hope.

The fundamental essence of transcendence lies, among other things, in the possibility of salvation. In analysing Marcel’s thoughts, Randall asks two questions: “salvation from what?” and “salvation for what?”. His answer to the first question is: “salvation from the prison of the self (egotism, narcissism, arrogance, avarice, envy, jealousy, etc.) and salvation from the finality of death” (Randall 1992, pp. 296–297). According to Randall, the second question lacks a satisfactory answer. Marcel declared that “salvation is indistinguishable from peace, it is a living peace…. This living peace, however, can be nothing but progress in love and truth” (Marcel 1960b, p. 205). The conclusion to be drawn from the analysis conducted throughout the article is that the second question, regarding the final purpose of Marcel’s concept of salvation understood in a larger meta-problematic sense, is the central category of Marcel’s discourse on hope, within which every experience of hope is perceived to contain an element of faith in a better reality, and one not necessarily related to any sort of religious belief (Knox 2011). According to Marcel, a belief in miracles is an act of faith signalling how mystery lies at the source of human beliefs. Through acts of communication and communion people can develop profound human bonds and intersubjective environments that can nourish personal hope.

## Conclusion

In summarizing the article’s main findings, we arrive at the following three conclusions. Firstly, despite terminological differences, the main purpose of Marcel’s phenomenology of hope, as was also demonstrated in the examples taken from the EOL studies, is to show the dynamic character of hope as a resourceful movement towards (the affirmation of) being. Marcel’s concept of hope which stems from the level of “Being” and “being” is also reflected in the EOL studies, in which patients tended to prefer hope as a verb to hope as a noun. The use of the noun would result in the objectification of hope, depriving it of its subjective aspect, i.e. the possibility of changing the specific situation in which the subject (the patient) is found. Objectified hope loses its mysterious dimension, thereby reducing the patient to a passive object undergoing therapeutic treatment. In this light, hope in the Marcelian sense of the word must be preserved as a profound personal and existential experience of medical care as it helps patients maintain serenity and a sense of quality of life.

Secondly, the important conclusion to be drawn from the analysis as a whole is that both the transcendent character of hope and the dialectic of hope and despair, can enrich and develop EOL care in terms of showing the importance of always keeping in mind the level of “being” or “Being” and the distinction between “being” and “having” as a means of interpersonal communication. As is reflected in empirical studies, Marcel’s phenomenology of hope can also encourage patients to improve social bonds with others as well as with themselves not only in a holistic sense but also from an existential perspective.

Thirdly, Marcel’s phenomenological method helps us observe recurrent categories and motifs that are inherent in hope as a mystery. This method also enabled Marcel to discuss hope from a metaphysical and ontological perspective that revealed its transcendent nature. To affirm this transcendent nature is to experience the exigency of being to which the individual aspires. The EOL research analysed in this paper tends to show that the mystery of hope is expressed implicitly by respondents as the most private and positive attitude in the first-person singular. The seed of Marcelian hope lies here, and can be unearthed through dialogue. As a consequence, by introducing Marcel’s phenomenology of hope into the clinical encounter, patients can be given the means of adapting to the difficult conditions associated with their disease and PC treatment. Additionally, by familiarizing with Marcel’s concept of hope, HCPs and empirical researchers can learn about the importance of his understanding of hope. This awareness can provide inspiration for qualitative research, e.g. the forming of questions in interviews that touch on the existential aspects of the patient’s situation in EOL care. Consequently, if studies are insufficiently aware this dimension of hope, it can be argued that they fail to cover an important aspect of hope for terminally ill patients in practice. Hope is necessary for creating meaning but domesticating it will dim its existential character and reduce it to desire and/or optimism. Marcel’s phenomenology of hope reminds us exactly of the need to reflect on the mysterious shades of hope.

## Footnotes

1 Neonatal Intensive Care Units.
